# Application of GLAD-PCR Assay for Study on DNA Methylation in Regulatory Regions of Some Tumor-Suppressor Genes in Lung Cancer

**DOI:** 10.3779/j.issn.1009-3419.2019.09.01

**Published:** 2019-09-20

**Authors:** N.A. Smetannikova, A.A. Evdokimov, N.A. Netesova, M.A. Abdurashitov, A.G. Akishev, E.V. Dubinin, P.I. Pozdnyakov, I.V. Vihlyanov, M.K. Nikitin, E.B. Topolnitsky, A.B. Karpov, S.A. Kolomiets, S.Kh. Degtyarev

**Affiliations:** 1 State Research Center of Virology and Biotechnology, Koltsovo, Russia; 2 EpiGene LLC, Novosibirsk, Russia; 3 Altai Regional Oncology Center, Barnaul, Russia; 4 Tomsk Regional Clinical Hospital, Tomsk, Russia; 5 Seversk Biophysical Research Centre, Seversk, Russia; 6 Regional Clinical Oncology Center, Kemerovo, Russia

**Keywords:** Lung neoplasms, DNA methylation, GLAD-PCR, Diagnostics, Epigenetics

## Abstract

Hypermethylation of the gene regulatory regions are common for many cancer diseases. In this work we applied GLAD-PCR assay for identificating of the aberrantly methylated RCGY sites in the regulatory regions of some downregulated genes in tissue samples of lung cancer (LC). This list includes *EFEMP1*, *EPHA5*, *HOXA5*, *HOXA9*, *LHX1*, *MYF6*, *NID2*, *OTX1*, *PAX9*, *RARB*, *RASSF1A*, *RXRG*, *SIX6*, *SKOR1* and *TERT* genes. The results of DNA samples from 40 cancer and 25 normal lung tissues showed a good diagnostic potential of selected RCGY sites in regulatory regions of *MYF6*, *SIX6*, *RXRG*, *LHX1*, *RASSF1A* and *TERT* genes with relatively high sensitivity (80.0 %) and specificity (88.0 %) of LC detection in tumor DNA.

## Introduction

Lung cancer (LC) is one of the major malignancies annually leading to more than 1.5 million cases of cancer death worldwide^[1, 2]^ and requiring an early diagnostics method.

The detection of methylation of RCGY sites in tumor-suppressor gene regulatory regions, which occurs in the tumor cell DNA at the initial stages of the disease, is one of the most promising diagnostic and prognostic tools. Such an aberrant methylation is typical for the most of sporadic cancers at early stages, constituting more than 90% of all malignant neoplasms^[3, 4]^.

It is well known that de novo DNA methylation, including abnormal DNA methylation in cancer cells, is performed by DNA methyltransferases Dnmt3a and Dnmt3b, which predominantly recognize RCGY site (whereas R is A or G, Y is T or C) and modify it with formation of R(5mC)GY sequence in both DNA strands^[5]^. Methyl-directed DNA endonuclease GlaI specifically recognizes and cleaves R(5mC)↓GY sites as indicated by the arrow^[6]^. Due to this unique specificity it is possible to use GlaI for identification of the tumor cell DNA.

Based on this enzyme we have previously developed a GLAD-PCR assay for detection of R(5mC)GY sequence in a definite position of human genome even at significant excess of DNA molecules with this non-methylated site (RCGY)^[7]^. The assay includes DNA hydrolysis with GlaI, ligation of the obtained DNA fragments with the specific adapter followed by real-time PCR with TaqMan probe and a genomic primer, which are complementary to target DNA fragment, and the second primer, which is complementary to the adapter sequence.

Recently, we used GLAD-PCR assay to analyze the tumor-suppressor genes methylation in DNA samples from colorectal cancer tissues^[8, 9]^. In the present work we apply GLAD-PCR assay to detect an aberrant methylation of RCGY sites in the regulatory regions of tumor-suppressor genes in DNA samples from tumor tissues in case of LC.

## Materials and methods

The study specimens were bronchopulmonary tumor DNA samples, obtained from 40 LC patients who had undergone surgery. The most of patients had squamous-cell lung carcinomas (*n*=19) or adenocarcinomas (*n*=13) with varying degrees of differentiation. In other cases small-cell carcinomas (*n*=2), large-cell carcinomas (*n*=2) and mixed carcinomas (*n*=2) (squamous-cell carcinoma combined with adenocarcinoma and large-cell carcinoma combined with neuroendocrine tumor) were diagnosed. Meanwhile five patients had clinical stage Ⅰ (T1-2аN0M0), 12 had stage Ⅱ (T1-3N0M0, T1-2N1M0), 18 had stage Ⅲ (T1-2N2M0, T3N1-2M0, T4N0-2M0, T1-4N3M0) and four patients were diagnosed with stage Ⅳ (distant metastases (M1) in any primary tumor size (T) and metastatic status of the regional lymph nodes (N)). In one case the malignant neoplasm was the local relapse of squamous-cell lung carcinoma with low differentiation degree.

As a control, we used DNAs from normal lung tissues, obtained from 25 LC patients during the surgery at a considerable distance from the tumor focus, on the resection line.

The aim of this study was preliminary selection of R(5mC)GY sites – potential epigenetic markers of lung cancer which will be further verified on wider number of samples obtained from blood cell-free DNA.

All participating patients voluntarily joined this study with the written informed consent.

The preservation procedure and storage conditions for tissue samples are described previously^[8]^. DNA isolation and purification were performed using the standard phenol-chloroform method^[10]^.

DNA samples were analyzed by GLAD-PCR assay as described previously in detail^[9]^.

To design specific primers and probes, we used the nucleotide sequences (human genome version GRCh38/hg38) from the GenBank database (http://ncbi.nlm.nih.gov/genbank), Vector NTI 11.5 software (Invitrogen, USA), and NCBI BLAST resource (http://blast.ncbi.nlm.nih.gov). A list of primers and probes is provided in Table 1. Hybrid primers, corresponding to the studied RCGY sites, were selected as described previously^[8, 9]^.

**1:** The genes selected for the study and a list of genomic primers and probes

Gene^a^	Protein name^a^	Chromosomal location^a^	Genomic primer/probe structure^b^
*EFEMP1*	EGF containing fibulin like extracellular matrix protein 1	2p16.1	FAM-CACCTCCACCTCCCTCTCTGCCCGA-BHQ1 CGAGAACTGCCCTGGACTGTGC
*EPHA5*	EPH receptor A5	4q13.1-q13.2	GAGGGAAGTAATCTGGAGGCTGCC FAM-TCCCGGAGACCGCTAGGCAACACTCA-BHQ1
*HOXA5*	homeobox A5	7p15.2	CGCAGGGCTGGGAGAAATGA FAM-CCCCCCTCTCTTCTCTGCCGCCC-BHQ1
*HOXA9*	homeobox A9	7p15.2	FAM-TGCTCACCATCCCTGTCGCAGAAGC-BHQ1 CCGACACGGCCGCTTCAGT
*LHX6*	LIM homeobox 6	9q33.2	FAM-CCTCACCGGGTTGCGACGAAGCC-BHQ1 GGCTCTGAACCTTCGATTCCTCCT
*MYF6*	myogenic factor 6	12q21.31	GCACCAGAGAAAATCCGGGAGGT FAM-CCCTCTCGGCCCAAGCATCCTATCC-BHQ1
*NID2*	nidogen 2	14q22.1	FAM-CCTGGAGTCCCGCCCCGCC-BHQ1 ccagccgaggggcctcag
*OTX1*	orthodenticle homeobox 1	2p15	CCAAAACCAGGGAACAAAACAACAAA FAM-CCCAGGCCCCAAACCCAGTTCCAA-BHQ1
*PAX9*	paired box 9	14q13.3	CATCCCCCCATCCCCAACAC FAM-CGCATTTTCCACCCTCCCCCGC-BHQ1
*RARB*	retinoic acid receptor beta	3p24	TTCAGAGGCAGGAGGGTCTATTC FAM-TCCCAGTCCTCAAACAGCTCGCATGG-BHQ1
*RASSF1A*	Ras association domain family member 1	3p21.31	ATGTCGGGGGAGCCTGAG FAM-TGCCAGCTCCCGCAGCTCAAT-BHQ1
*RXRG*	retinoid X receptor gamma	1q22-q23	GCCGCCGTCACCGCTACT FAM-CCACCGCCGTCGCTGCTGC-BHQ1
*SIX6*	SIX homeobox 6	14q23.1	GTGGCAACTACCGCGAGCTCT FAM-ATCCTGGAAAACCACAAGTTCACCAAGGA-BHQ1
*SKOR1*	SKI family transcriptional corepressor 1	15q23	GCCGAGCGGGTTCCAGGA FAM-CCTCTTCCCCCTCCTCGGTTCCGGT-BHQ1
*TERT*	telomerase reverse transcriptase	5p15.33	FAM-CTCCCTGCAACACTTCCCCGCGA-BHQ1 AAAGAGAAATGACGGGCCTGTGTC
^a^ Gene symbol, protein name, and chromosomal location are in accordance with the approved guidelines from the HUGO Gene Nomenclature Committee (http://www.genenames.org). b Structure of the direct or reverse genomic primer is given ahead or after probe, respectively.			

The statistical analysis of experimental data was performed using the MedCalc 15.11 software (MedCalc Software, Ostend, Belgium). According to the quantification cycle (Cq) values obtained for each studied RCGY site, receiver operating characteristic (ROC) curves with 95 % confidence interval (CI) were determined to assess the assay sensitivity and specificity with an area under the ROC curve (AUC) being estimated nonparametrically. AUC is the cumulative indicator of the marker diagnostic efficacy (for the "perfect" test AUC = 1) ^[11]^.

## Results

The determination of candidate tumor-marker genes in lung cancer. When searching for LC epigenetic markers, DNA samples from the tumor and normal lung tissues are mainly examined. The comparison of gene methylation status in tumor and normal cells enables to identify potential epigenetic tumor markers that are much more frequently methylated in malignant transformation.

As follows from the recent studies, a number of candidate epigenetic LC determinants have been identified using bisulfite DNA conversion method. We chose 15 tumor-suppressor genes in regulatory regions of which we studied methylation of RCGY sites using GLAD-PCR assay. This list includes *MYF6*, *SIX6* и *RARB* РЛ^[12]^, *NID2* и *RASSF1A*^[13]^, *TERT* и *RASSF1А*^[14]^, *EFEMP1*^[15]^, *EPHA5*^[16]^, *HOXA5*^[17]^, *HOXA9*^[18]^, *LHX6*^[19]^, *OTX1*^[20]^, *PAX9*^[21]^, *RXRG*^[22]^ и *SKOR1* genes ^[23]^.

Identification of RCGY sites in DNA from LC tissues suitable for GLAD-PCR analysis. Screening of the mostly methylated RCGY sites in regulatory regions of tumor-suppresor genes was carried out as described earlier^[8, 9]^ using 10 DNA samples. For each RCGY site in the studied DNA region (~200 nucleotides from the genomic primer hybridization site) we used corresponding hybrid primer. A criterion of the site selection was the lowest value of the threshold cycle (Cq) obtained in GLAD-PCR assay.

The selected R(5mC)GY sites and corresponding hybrid primers, which were used for further study of the samples DNA collection (both LC and normal tissue samples), are provided in Table 2.

**2:** Studied genes with indication of the target RCGY site, its location and a structure of corresponding hybrid primer selected for GLAD-PCR assay

Gene	Target site	Site location^a^	Hybrid primer^b^
*EFEMP1*	GCGC	chr3: 55923479–55923482	CCTGCTCTTTCATCGGCGC
*EPHA5*	GCGC	chr4: 65670704–65670707	CCTGCTCTTTCATCGGCCT
*EPHA5*	GCGT	chr4: 65670706–65670709	CCTGCTCTTTCATCGGCGC
*HOXA5*	GCGT	chr7: 27143017–27143020	CCTGCTCTTTCATCGGCGA
*HOXA5*	GCGC	chr7: 27144072–27144075	CCTGCTCTTTCATCGGCGA
*HOXA9*	GCGC	chr7: 27174676–27174679	CCTGCTCTTTCATCGGCCC
*LHX6*	GCGC	chr9: 122219596–122219599	CCTGCTCTTTCATCGGCGT
*MYF6*	gcGC	chr12: 80708733–80708736	CCTGCTCTTTCATCGGCGC
*NID2*	gcGC	chr14: 52069060–52069063	CCTGCTCTTTCATCGGCCG
*OTX1*	GCGC	chr2: 63057659–63057662	CCTGCTCTTTCATCGGCGG
*PAX9*	GCGC	chr14: 36663813–36663816	CCTGCTCTTTCATCGGCGA
*RARB*	ACGC	chr3: 25428374–25428377	CCTGCTCTTTCATCGGTTC
*RASSF1A*	gcGC	chr3: 50340715–50340718	CCTGCTCTTTCATCGGCGT
*RXRG*	GCGC	chr1: 165445276–165445279	CCTGCTCTTTCATCGGCCG
*SIX6*	GCGC	chr14: 60509991–60509994	CCTGCTCTTTCATCGGCGG
*SKOR1*	ACGC	chr15: 67821744–67821747	CCTGCTCTTTCATCGGTTC
*TERT*	GCGT	chr5: 1295645–1295648	CCTGCTCTTTCATCGGCGG
^a^ Site locations are given in accordance with the recent human genome assembly GRCh38/hg38; b 3'-terminal tetranucleotide sequence of hybrid primer, which is complemented to the genomic sequence at the point of GlaI hydrolysis, is underlined.			

GLAD-PCR assay of RCGY sites in DNAs from the clinical samples. GLAD-PCR assay of selected sites in DNAs, obtained from LC samples (*n*=40) and normal lung tissue (*n*=25) was performed in triplets. There was 3 ng of DNA (~10^3^ copies of the studied gene region) in the reaction mixture. Figure 1 presents the diagrams of the average values of threshold amplification cycles (Cq) obtained by GLAD-PCR analysis of RCGY sites located in LC tumor-suppressor genes.

**1:**

GLAD-PCR assay of selected RCGY sites. Cq values (with the standard deviation range) are given for DNA samples which are numerated below each diagram (T: tumor tissue, N: normal tissue).

The statistical analysis of the results of GLAD=PCR assay (Figure 2 and Table 3) allows to draw a conclusion about the good diagnostic characteristics of the RCGY sites in tumor-suppressor genes *MYF6*, *SIX6*, *RXRG*, *LHX1*, *RASSF1A* and *TERT*: obtained AUC values for these markers are in 0.727-0.805 range^[11]^).

**2:**
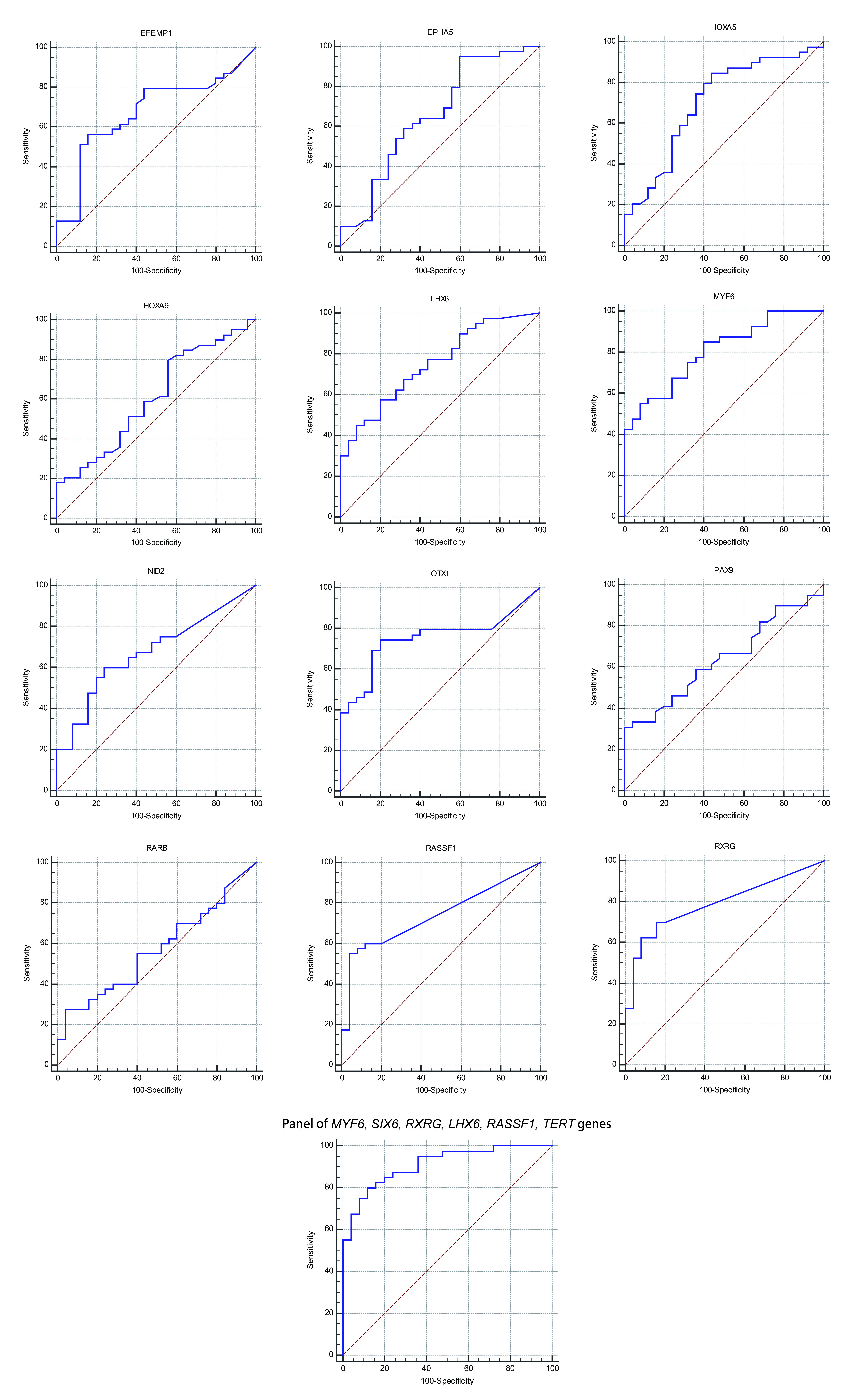
ROC-curves for GLAD-PCR analysis of selected RCGY sites in LC versus normal lung tissues. Resulting sensitivity, specificity, area under the ROC curve (AUC) with standard error and 95 % confidence interval (CI) are summarized in Table 3.

**3:** Receiver operating characteristics of GLAD-PCR assay of selected RCGY sites (sorted by AUC values) for LC versus normal lung tissue

Gene	Number of detected LC samples/total number of LC samples	Sensitivity, %	Number of negative controls/total number of normal lung tissue controls	Specificity, %	AUC – Area under curve (standard error)	95%CI
*MYF6*	30/40	75.0	18/25	72.0	0.805 (0.053)	0.688–0.893
*SIX6*	26/40	65.0	22/25	88.0	0.795 (0.059)	0.677–0.885
*RXRG*	23/40	57.5	23/25	92.2	0.772 (0.051)	0.651–0.867
*LHX6*	31/40	77.5	15/25	60.0	0.746 (0.061)	0.622–0.845
*RASSF1A*	22/40	55.0	24/25	96.0	0.733 (0.056)	0.608–0.835
*TERT*	20/40	50.0	24/25	96.0	0.727 (0.062)	0.602–0.830
*HOXA5*	28/39	71.8	16/25	64.0	0.697 (0.069)	0.570–0.806
*EFEMP1*	23/39	59.0	20/25	80.0	0.689 (0.069)	0.561–0.799
*OTX1*	21/29	72.4	18/25	72.0	0.666 (0.077)	0.524–0.788
*NID2*	21/40	52.5	21/25	84.0	0.666 (0.066)	0.539–0.779
*EPHA5*	35/39	89.7	10/25	40.0	0.642 (0.075)	0.512–0.758
*SKOR1*	10/29	34.5	25/25	100	0.639 (0.071)	0.497–0.765
*PAX9*	13/39	33.3	24/25	96.0	0.634 (0.069)	0.505–0.751
*HOXA9*	8/39	20.5	25/25	100	0.604 (0.073)	0.474-0.724
*RARB*	11/40	27.5	24/25	96.0	0.558 (0.072)	0.429–0.681
Panel of *MYF6*, *SIX6*, *RXRG*, *LHX6*, *RASSF1A*, *TERT* genes	32/40	80.0	22/25	88.0	0.911 (0.034)	0.814–0.967

For these six LC tumor-markers, the total diagnostic characteristics were calculated using the logistic regression method, which enables the analysis of the relationship between several independent variables (the values of the threshold PCR cycles for the studied DNA samples for each marker) and dependent one (DNA sample from tumor or normal tissue). As follows from the Figure 2 and Table 3, the tumor-marker combination significantly increases sensitivity and specificity of LC diagnosis: AUC value evaluates 0.911.

## Discussion

Nowadays, a large number of LC epigenetic determinants have been described which are characterized by the differences in DNA methylation status between tumor and normal lung tissue cells.

Thus Zhao Y *et al*. investigated 101 tumors and 30 normal lung tissue samples and demonstrated that regulatory regions of MYF6, SIX6 and RARB tumor-suppressor genes are the best epigenetic LC markers^[12]^. Another group investigated 103 tumors and 23 normal lung tissue samples and suggested using NID2 and RASSF1A genes as LC tumor-markers^[13]^. Based on the analysis of DNA isolated from bronchial swabs of 333 LC patients and 323 healthy donors, Nikolaidis G. *et al*. identified a tumor-marker combination, including the regulatory regions of the *TERT* and *RASSF1A* genes, as the most informative for LC diagnosis^[14]^. Other authors characterized epigenetic LC markers within *EFEMP1*^[15]^, *EPHA5*^[16]^, *HOXA5*^[17]^, *HOXA9*^[18]^, *LHX6*^[19]^, *OTX1*^[20]^, *PAX9*^[21]^, *RXRG*^[22]^ и *SKOR1*^[23]^ tumor-suppressor genes.

It is possible that such a variety of epigenetic markers is explained by the histological heterogeneity of LC, which is subdivided into two main types: small-cell lung carcinoma and non-small-cell lung carcinoma (about 15 % and 85 % of all cases, respectively). In turn, non-small cell LC includes three subtypes: squamous-cell carcinoma, adenocarcinoma and large-cell carcinoma, which occur in ratio close to 2.5:4:1^[24]^.

GLAD-PCR opportunities for the diagnostics of lung cancer. The results obtained in this work demonstrate that GLAD-PCR analysis enables to determine R(5mC)GY sites in regulatory regions of tumor-suppressor genes in DNA samples isolated from LC tissue.

We have previously shown that GLAD-PCR assay can be used to analyze any complex DNA mixture, for example, DNA isolated from a patient's blood containing nucleic acids both from tumor and normal cells (vascular cells, connective tissue cells, *etc*.). The site methylation degree determines the threshold cycle value (Cq) in real-time PCR^[7]^.

The algorithm used for the ROC analysis of experimental data makes it possible to evaluate the reliability of the difference in the methylation status of tumor-suppressor genes in the DNA of tumor and normal cells and to identify potential epigenetic LC markers. The most promising markers (having maximum AUC values) are the target sites RCGY sites in regulatory regions of *MYF6*, *SIX6*, *RXRG*, *LHX1*, *RASSF1A*, and *TERT* genes.

A comprehensive study of gene panel significantly increases the sensitivity of the test (the total value is 80.0 %) in comparison with the analysis of individual markers (Figure 2 and Table 3).

Subsequently, in order to improve the diagnostic efficiency of the epigenetic LC marker panel, we intend to study a larger number of RCGY sites in other tumor-suppressor genes and a greater number of tissue samples belonging to different lung cancer subtypes. It will possibly change the panel composition.

## Conclusion

GLAD-PCR assay enables to identify R(5mC)GY sites, arising from the aberrant methylation of regulatory regions of tumor-suppressor gene in LC tissue samples. In this study of DNA sample from clinical specimens we have shown a good diagnostic efficacy of the epigenetic LC markers panel including the RCGY sites in regulatory regions of *MYF6*, *SIX6*, *RXRG*, *LHX1*, *RASSF1A* and *TERT* genes: total sensitivity and specificity are 80.0 % and 88.0 %, respectively.

The present study was supported by the Skolkovo Foundation (Under agreement NO. G102/16 06.12.2016 г.).

Authors declare lack of the possible conflicts of interests.
